# UV-B-induced signaling events leading to enhanced-production of catharanthine in *Catharanthus roseus *cell suspension cultures

**DOI:** 10.1186/1471-2229-7-61

**Published:** 2007-11-07

**Authors:** Shilpa Ramani, Jayabaskaran Chelliah

**Affiliations:** 1 Department of Biochemistry, Indian Institute of Science, Bangalore, 560012, India

## Abstract

**Background:**

Elicitations are considered to be an important strategy towards improved *in vitro *production of secondary metabolites. In cell cultures, biotic and abiotic elicitors have effectively stimulated the production of plant secondary metabolites. However, molecular basis of elicitor-signaling cascades leading to increased production of secondary metabolites of plant cell is largely unknown. Exposure of *Catharanthus roseus *cell suspension culture to low dose of UV-B irradiation was found to increase the amount of catharanthine and transcription of genes encoding tryptophan decarboxylase (*Tdc*) and strictosidine synthase (*Str*). In the present study, the signaling pathway mediating UV-B-induced catharanthine accumulation in *C. roseus *suspension cultures were investigated.

**Results:**

Here, we investigate whether cell surface receptors, medium alkalinization, Ca^2+ ^influx, H_2_O_2_, CDPK and MAPK play required roles in UV-B signaling leading to enhanced production of catharanthine in *C. roseus *cell suspension cultures. *C. roseus *cells were pretreated with various agonists and inhibitors of known signaling components and their effects on the accumulation of *Tdc *and *Str *transcripts as well as amount of catharanthine production were investigated by various molecular biology techniques. It has been found that the catharanthine accumulation and transcription of *Tdc *and *Str *were inhibited by 3–4 fold upon pretreatment of various inhibitors like suramin, N-acetyl cysteine, inhibitors of calcium fluxes, staurosporine *etc*.

**Conclusion:**

Our results demonstrate that cell surface receptor(s), Ca^2+ ^influx, medium alkalinization, CDPK, H_2_O_2 _and MAPK play significant roles in UV-B signaling leading to stimulation of *Tdc *and *Str *genes and the accumulation of catharanthine in *C. roseus *cell suspension cultures. Based on these findings, a model for signal transduction cascade has been proposed.

## Background

*C. roseus *produces terpenoid indole alkaloids (TIAs) as a part of its secondary metabolism. TIAs provide protection against microbial infection, herbivores and abiotic environmental stresses such as UV irradiation [1,2]. Some of the TIAs are of pharmaceutical importance such as the antitumor dimeric alkaloids, vincristine and vinblastine, and the anti-hypertensive monomeric alkaloids, ajmalicine and serpentine [3]. The anti-tumor dimeric alkaloids, which accumulate in the leaves of *C. roseus*, are composed of catharanthine and vindoline monomers and are exclusively found in *C. roseus *plants. In plants, the dimeric alkaloids and the monomer catharanthine accumulate in low amounts whereas the monomer vindoline accumulates at a relatively higher level [4,5]. *C. roseus *cell cultures have been investigated as alternative means of production of terpenoid indole alkaloids, but they failed to produce vindoline [6]. Therefore, it has been considered desirable to produce the dimers by coupling catharanthine obtained from cell cultures with vindoline obtained from the cultivated plants. The production of catharanthine by *C. roseus *cell cultures has been one of the most extensively explored areas of plant cell culture and is still limited due to the low yield [7].

Elicitations are considered to be an important strategy towards improved *in vitro *production of secondary metabolites. In cell cultures, biotic and abiotic elicitors have effectively stimulated the production of plant secondary metabolites [8]. Fungal elicitors have been widely tested for elicitation of catharanthine production in various *C. roseus *cells [5,9]. However, molecular basis of elicitor-signaling cascades leading to increased production of secondary metabolites of plant cell is largely unknown. It is known that receptor proteins that bind elicitors generate signals that are transmitted to the sites of gene expression via different components, such as Ca^2+^/ion fluxes, medium alkalinization and cytoplasmic acidification, oxidative burst, jasmonate and nitric oxide *etc*. [8]. Many CDPKs and MAPKs have been identified to play a role in defense responses and also secondary metabolite production [10].

The effect of UV-B irradiation on expression of TIA biosynthetic genes, *Tdc *and *Str*, and catharanthine production has been reported previously in *C. roseus *leaves[11-13]. The transcription factor GT-1 binds to the promoter region of *Tdc in vitro*. The functional importance of GT-1 in the induction of *Tdc *expression by UV light has been demonstrated by point mutations in the GT-1 binding site [14]. However, the molecular basis of UV-B signaling cascades leading to the induction of expression of *Tdc *and *Str *genes and the production of TIAs is largely unknown. It has been observed that the polypeptide wound signal, systemin- specific cell surface receptors initiate a signal transduction cascade upon UV-B irradiation in *L. peruvianum *cell suspension cultures [15]. In the present study, the signaling pathways mediating UV-B-induced catharanthine accumulation in *C. roseus *suspension cultures were investigated. UV-B induced alkalinization of the culture medium, generation of hydrogen peroxide, activation of CDPK and MBPK as well as accumulation of catharanthine and stimulation of transcription of *Tdc *and *Str *genes were studied. Inhibitors of binding of ligand-cell surface receptors, protein kinases and phosphatases, calcium fluxes and H_2_O_2 _were used to dissect the UV-B signaling cascade.

## Results

### Alkalinization of *C. roseus *cell-suspension medium in response to UV-B irradiation and its inhibition by suramin

**Figure 1:**
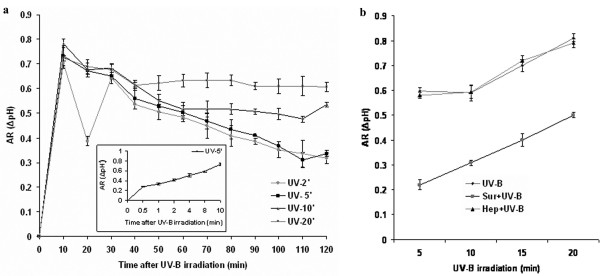
Medium alkalinization of *C. roseus *suspension cultured cells in response to UV-B irradiation and its inhibition by suramin. (**a**)Six-day-old cell suspension cultures were either irradiated with UV-B or left untreated for various periods of time and the pH of the medium was measured at the times indicated after the start of irradiation. Alkalinization response (AR or Δ pH) was measured as described in materials and methods. Inset: Early medium alkalinization response to 5 min of UV-B irradiation (**b**) Inhibition by suramin of UV-B-induced medium alkalinization. Cells were pre-treated with 1 mM suramin or 1 mM heparin for 10 min prior to irradiation with different doses of UV-B, and as control, cells were irradiated with UV-B alone and the pH of the medium was measured after 10 min. The increase in medium pH (Δ pH) is indicated as the difference between the pH at time 0 and at 10 min. Bars represent the means ± SD (n = 6).

Medium alkalinization an early event occurring in elicitor- treated plant cell cultures, has been used as a marker of elicitor responses in studying elicitor-binding sites in plant cells [16]. Medium alkalinization is thought to result from elicitor/stress-induced depolarization of the plasma membrane and subsequent K^+^/H^+ ^exchange with Ca^2+ ^influx/Cl ^- ^efflux [16]. To determine whether medium alkalinization is involved in UV-B signal transduction as an early event, six-day-old cells were exposed to UV-B irradiation for various time periods (2, 5, 10 or 20 min) and extracellular pH changes were measured in the cell-suspension medium for 120 min. As shown in Figure 1a, the effect of UV-B on medium alkalinization was not dose-dependent. However, the kinetics and intensity of this response were dependent on their respective exposure times. *C. roseus *cells showed a rapid increase in the medium pH after UV-B irradiation peaking at 10 min with an increase of about 0.7 units in 5-min irradiated cells (Fig 1a inset). The other doses of UV-B irradiation on cells did cause an increase in AR, but in all cases the pH of the medium decreased but never returned back to baseline levels even after 24 h, which probably could be due to the damage caused by prolonged exposure to UV-B (data not shown). In the cells irradiated with 2 and 5 min of UV-B however, the pH of the medium returned to baseline by 300 min (data not shown). Cell viability when checked after 24 h of irradiation showed that irradiation with UV-B for 2 min and 5 min did not cause cell death (98% cell survival as visualized by florescein diacetate/propidium iodide staining); however, irradiation for longer than 5 min caused 80 – 100 % cell death (data not shown). We have therefore used 5 min of UV-B as the standard irradiation time for all further experiments.

Suramin is known to bind with cell surface components such as the systemin receptor [17] and interfere with the signaling events and this system is affected by UV-B irradiation in *L. peruvianum *cells [15]. Since UV-B irradiation of *C. roseus *cells caused alkalinization of the medium, we investigated whether suramin could inhibit the UV-B-induced medium alkalinization. The results show that the UV-B-induced alkalinization was inhibited by suramin (Figure 1b). Suramin inhibited alkalinization of the growth medium for all exposure times of UV-B irradiation. Heparin, which is similar to suramin in possessing polysulfonated groups, had no effect on alkalinization of the medium induced by UV-B irradiation.

### UV-B-induced H_2_O_2 _production and involvement of protein kinases in UV-B-induced H_2_O_2 _production

**Figure 2:**
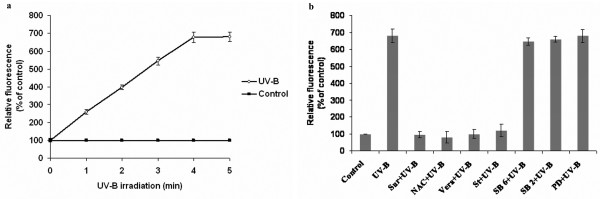
Production of ROS in *C. roseus *suspension cultured cells in response to UV-B irradiation. (**a**) A time course of UV-B induced ROS production. Six-day-old cell suspension cultures were irradiated by UV-B for different times and 2.5 μM DCFH-DA was added. The ROS production was measured after 15 min as a difference in the fluorescence intensity between the UV-B-irradiated and untreated controls. Bars represent means ± SD (n = 3). (**b**) Effect of various inhibitors on UV-B induced ROS production. Six-day-old cell suspension cultures were treated with 1 mM suramin (Sur), 10 mM N-acetyl cysteine (NAC), 0.5 μM verapamil (Vera), 10 nM staurosporine (St), 40 nM SB 600125, a JNK inhibitor (SB6), 70 nM SB 203580, a P38 inhibitor (SB2) and 5 μM PD 98059, an ERKK inhibitor (PD) for 10 min prior to UV-B irradiation of 5 min and 2.5 μM DCFH-DA was added to the treated cultures. The ROS produced was measured as above.

The oxidative burst, a rapid consumption of oxygen and production of reactive oxygen species (ROS) such as H_2_O_2_, is a typical early event in plant defense responses [18,19]. With 5 min of UV-B irradiation of *C. roseus *cells H_2_O_2 _production increased six-fold compared to control cells (Fig 2a). We next examined effects of suramin, an inhibitor of G-protein inhibitor, N-acetyl cysteine, a putative ROS scavanger, verapamil, a calcium channel blocker and staurosporine, a serine-threonine kinase inhibitor, SB 203580, a P38 MAPK inhibitor, PD 98059, an ERKK inhibitor and SB 600125 JNK inhibitor. The UV-B- induced H_2_O_2 _production was suppressed by all the inhibitors except the MAPK cascade inhibitors (Fig 2b). This indicated that upon receiving the UV-B signal by a putative receptor in *C. roseus *cells, calcium influx and activation of serine/threoine kinases are required to induce H_2_O_2 _production. However, activation of the MAPK cascade occurs downstream of H_2_O_2 _production.

### Activation of protein kinases in response to UV-B irradiation in *C. roseus *suspension cell cultures

Many protein kinases are known to respond to both biotic and abiotic stresses. Two kinases, MAPKs and CDPKs, have been implicated to play pivotal roles in response to diverse stimuli [17,20]. Previous studies have demonstrated that *C. roseus *cells also respond to UV-B irradiation by expressing biosynthetic genes and production of TIAs [13]. To establish a functional link between these processes, we first examined the possible activation of MAPK and CDPK in cells irradiated with UV-B. MBP is known to be a conventional MAPK substrate and MAPK homologs also have MBP kinase activity [21]. To determine if a MAPK is associated with the UV-B signaling the activation of MBP kinase was investigated

**Figure 3:**
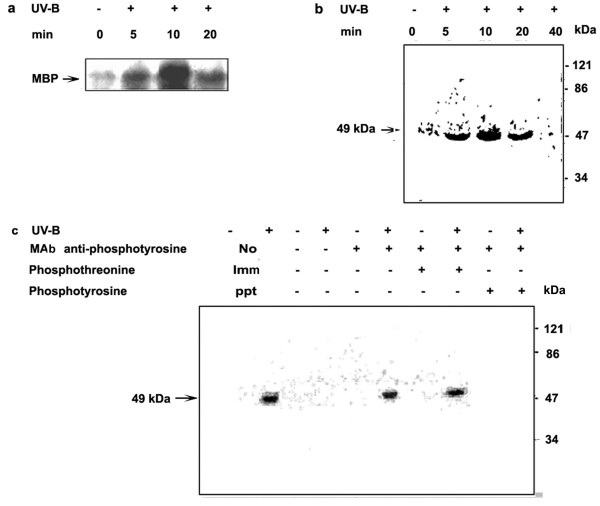
Activation of Myelin Basic Protein Kinase (MBPK) activity by UV-B irradiation in *C. roseus *suspension cultured cells. Six-day-old cell suspension cultures were irradiated for 5 min with UV-B light (+) or left un-irradiated (-) as a control. Cells were harvested at the indicated time periods, crude extracts were prepared, and MBPK activity in the cell extracts was assayed using MBP as a substrate as described in materials and methods. (**a**) MBPK activity was carried out with an *in vitro *phosphorylation assay. The reaction mixtures were resolved by SDS 10% (w/v) polyacrylamide gel electrophoresis and the phosphorylated MBP was visualized by autoradiography. (**b**) MBPK activity in the cell extracts was determined by in-gel kinase assay with MBP as a substrate. Autoradiogram represents in-gel phosphorylation of MBP. (**c**) Detection of MBPK activity in immunoprecipitates from cell extracts using the anti-phosphotyrosine antibody. Lane 1 and 2 represent cell extracts subjected to in-gel kinase assay directly without immunoprecipitation. Lane 3 to 10 indicate the cell extracts subjected to immunoprecipitation with a monoclonal antibody specific for phosphotyrosine and the MBPK activity of the immunoprecipitates assayed by in-gel kinase assay. The phosphorylated MBP was visualized by autoradiography. Phosphotyrosine and phosphothreonine were used as competitor substrates to demonstrate the specificity of the antibody. Symbols (-) and (+) represent, untreated and treated of the indicated treatment.

*C. roseus *cell suspensions were exposed to UV-B irradiation for 5 min and the cells were then assayed for MBPK and CDPK activities for different time periods. *In vitro *assays were performed in the cell extracts prepared from UV-B irradiated and control *C. roseus *cells. Figure 3a indicates that MBPK activity in UV-B irradiated cells significantly increased by 5 min and peaked at 10 min after UV-B irradiation. The MBPK activity remained high and above the control levels even at 20 min following irradiation. In order to identify specific MBPK activity induced by UV-B, an in-gel kinase assay was carried out. Figure 3b shows that in UV-B irradiated cells, the activity of one major protein kinase could be detected in the polyacrylamide gel containing MBP. From the mobility of the MBPK activity band during SDS-PAGE, the apparent molecular mass of the enzyme was estimated to be approximately 49 kDa. The 49-kDa MBPK activity increased by UV-B irradiation in cells compared with that of the un-irradiated control. The maximum MBPK activity was observed at 10 min after UV-B treatment. In all the *in vitro *experiments carried out with MBP as substrate, the phosphorylation peaked at 10 min; these results were consistently obtained when the experiments were repeated with different batches of cells. Therefore, in all further experiments the MBPK activity was assayed at 10 min after irradiation.

To further characterize the MBPK activity induced by UV-B, immunoprecipitation and in-gel kinase assays were used. The protein extracts were incubated with anti-phosphotyrosine monoclonal antibody and immunoprecipitated with protein A-agarose. The immunoprecipitated proteins were separated on a SDS-polyacrylamide gel containing MBP as a substrate and MBPK activity was assayed in the gel in the presence of ^32^P- ATP. As shown in Figure 3c, a 49 kDa protein kinase was again detected in the immunoprecipitate from UV-B-irradiated cells. Co-incubation with phosphotyrosine prevented immunoprecipitation of the 49 kDa protein kinase with anti-phosphotyrosine antibody, but co-incubation with phosphothreonine did not. These results indicate that only phosphotyrosine and not phosphothreonine could act as a competitor during immunoprecipitation, showing that MBP phosphorylating kinase was specifically phosphorylated on a tyrosine residue. Till date MAPK are the only known plant kinases to be phosphorylated on tyrosine residues.

**Figure 4:**
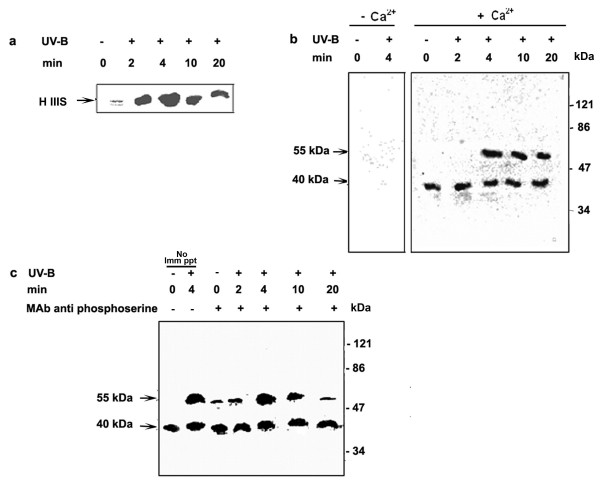
Activation of CDPK in *C. roseus *suspension cultured cells in response to UV-B irradiation. Six-day-old cell suspension cultures were irradiated for 5 min with UV-B light (+) or left un-irradiated (-) as a control. Cells were harvested at the indicated time periods, crude extracts were prepared, and the activity of CDPK in the cell extracts was assayed using histone IIIS as a substrate as described in materials and methods. (**a**) CDPK was assayed with an *in vitro *phosphorylation assay. The reaction mixtures were resolved by SDS 10% (w/v) polyacrylamide gel electrophoresis and subjected to autoradiography. (**b**) CDPK activity in the cell extracts were determined by in-gel kinase assay with histone IIIS as substrate in the presence and absence of calcium. Autoradiogram represents in-gel phosphorylation of histone IIIS. Arrows show the molecular masses of two detected CDPK bands (**c**) Detection of CDPK activity in immunoprecipitates from cell extracts using anti-phosphoserine antibody. Lane 1 and 2 represent cell extracts subjected to in-gel kinase assay directly without immunoprecipitation. Lane 3 to 7 indicate the cell extracts subjected to immunoprecipitation with a monoclonal antibody specific for phosphoserine and the CDPK activity of the immunoprecipitates assayed by in-gel kinase assay. The phosphorylated histone IIIS was visualized by autoradiography. Symbols (-) and (+) represent, untreated and treated of the indicated treatment. Arrows show the molecular masses of two detected CDPK bands.

Calcium dependent protein kinases (CDPKs) belong to the unique family of calcium-regulated kinases and histone IIIS was one of the best exogenous substrates for assaying CDPKs [22]. To characterize the kinase(s) induced by UV-B, the activities were assayed using histone IIIS as a substrate in protein extracts from cells irradiated with UV-B, as well as the controls. The protein extracts from 5-min UV-B irradiated cells, assayed in the presence of calcium using histone IIIS as substrate showed that, the kinase activity increased significantly peaking at 4 min after UV-B irradiation and remained high even at 20 min after UV-B irradiation (Figure 4a). The protein extracts from 5-min UV-B irradiated cells assayed by in- gel kinase assay in the absence and presence of calcium using histone IIIS as substrate demonstrated that the phosphorylation of histone IIIS was calcium dependent in both UV-B irradiated and un-irradiated cells (Figure 4b). CDPK activities were identified at two positions with an apparent molecular weight of 55 kDa and 40 kDa. One of the CDPK activated had an apparent molecular weight of 40 kDa and was constitutive, as it was observed to phosphorylate histone IIIS to a similar extent in both un-irradiated and irradiated cells whereas the 55 kDa kinase activity showed UV-B dependence and peaked at 4 min. Therefore, the phosphorylation of histone IIIS observed *in vitro *experiments was both due to the activities of the 55 and 40 kDa kinases. CDPKs being serine-threonine kinases are phosphorylated on both serine and threonine residues. To differentiate between MBP kinase detected in our experiments and the histone IIIS kinase, we used anti-phosphoserine monoclonal antibody for immunoprecipitation followed by a pull down with Protein A-agarose and assayed by in-gel kinase assay containing histone IIIS as substrate. Figure 4c shows that the 55 and 40 kDa kinases identified by in-gel kinase assay in Figure 4b were both phosphorylated on serine residues and that the activity of 40 kDa kinase was constitutive in our cell cultures. In all the *in vitro *experiments carried out with histone IIIS as substrate, the phosphorylation peaked at 4 min. These results were consistently obtained when the experiments were repeated with different batches of cells. Therefore, in all further experiments the CDPK activity was assayed at 4 min after irradiation.

### UV-B-induced MBPK and CDPK activities, *Tdc *and *Str *gene expression and catharanthine accumulation are inhibited by suramin

**Figure 5:**
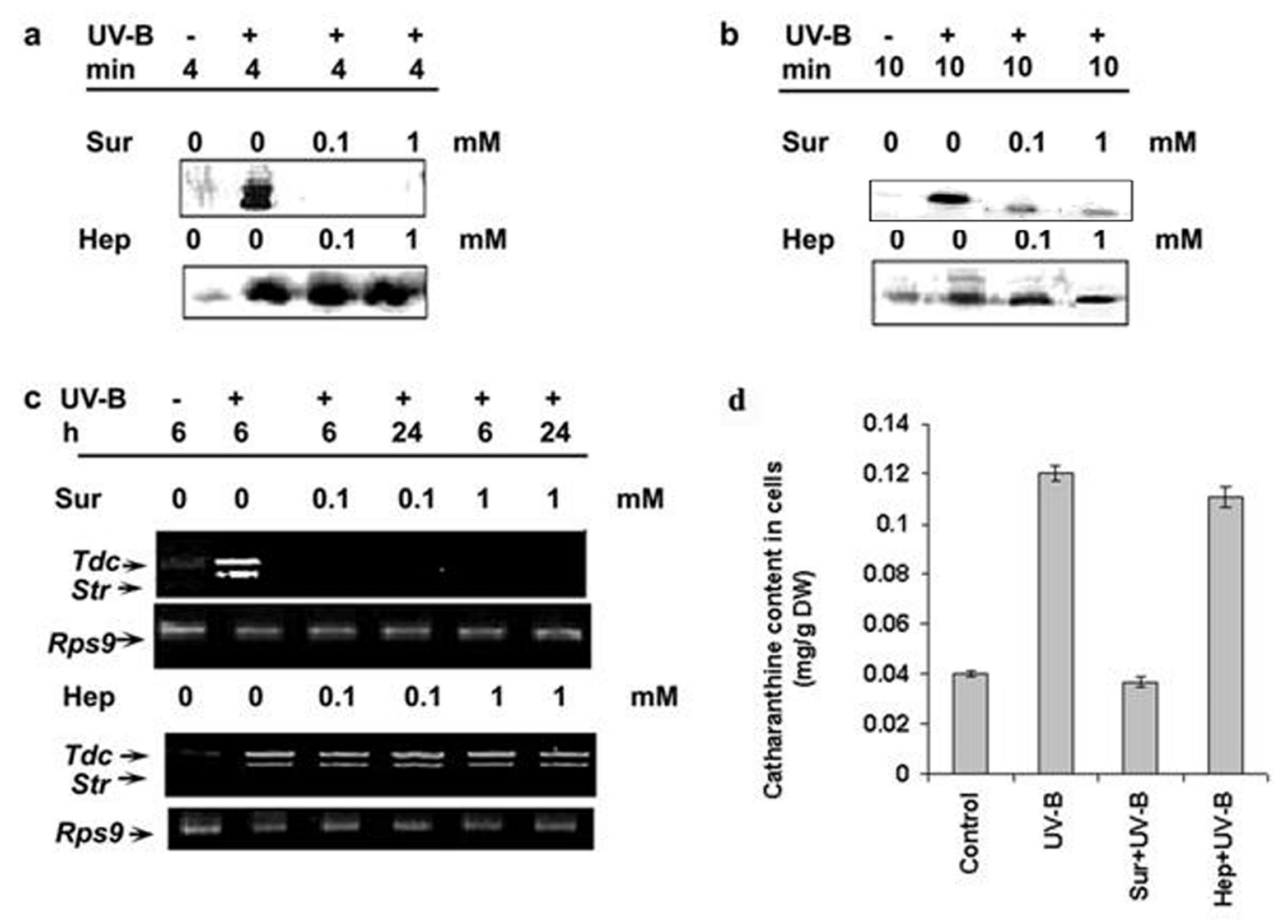
Effects of suramin and heparin on UV-B-induced CDPK activity (**a**), MBPK activity (**b**), *Tdc *and *Str *gene expression (**c**) and accumulation of catharanthine (**d**) in cell suspension cultures of *C. roseus*. Six-day-old cell suspension cultures were pre-treated with suramin (Sur) or heparin (Hep) at the indicated concentrations and were irradiated with UV-B for 5 min. As control one set of cells was irradiated with UV-B alone or left un-irradiated and the crude extracts from all cells were prepared at the indicated times and assayed for the phosphorylation of H IIIS (**a**) and MBP (**b**) under standard conditions as described in materials and methods. A second set of cells was similarly treated and the total RNA was isolated at the indicated times and analyzed for the transcript levels of *Tdc *and *Str *by RT-PCR (**c**). The third set of cells were pretreated with the highest concentration of inhibitor previously used followed by 5 min of UV-B irradiation. After treatment, cells were collected after 48 h and catharanthine content was determined by HPLC (**d**). These experiments were performed in triplicates and repeated at least twice. Error bars represent mean ± SD (n = 3).

Since the UV-B-induced early cellular responses *viz*., medium alkalinization and ROS production were inhibited by suramin, we investigated whether suramin could inhibit the UV-B induced other cellular responses related to synthesis of TIAs. When the cells were pretreated for 10 min with 0.1 and 1 mM suramin concentrations and subsequently irradiated with UV-B for 5 min, the UV-B-induced MBPK and CDPK activities, accumulation of *Tdc *and *Str *transcripts and catharanthine was strongly inhibited (Figure 5a–d). However, the UV-B-induced MBPK activity could not be completely inhibited by suramin. To rule out the possibility that the inhibitory effects of suramin on responses triggered by UV-B are not due to the unspecific binding to cell surface components, we used heparin a structurally similar molecule *viz*., heparin that possesses sulfonic acid groups similar to that of suramin for inhibition of UV-B responses. Figure 5a–d shows that heparin at both 0.1 and 1 mM concentrations had no effect on any of the UV-B mediated signaling events investigated demonstrating that the effect of suramin was specific under UV-B irradiated conditions. These data indicate that suramin-sensitive cell surface receptor may participate in the UV-B responses.

### Role of Ca^2+ ^in UV-B induced responses in *C. roseus *cells

**Figure 6:**
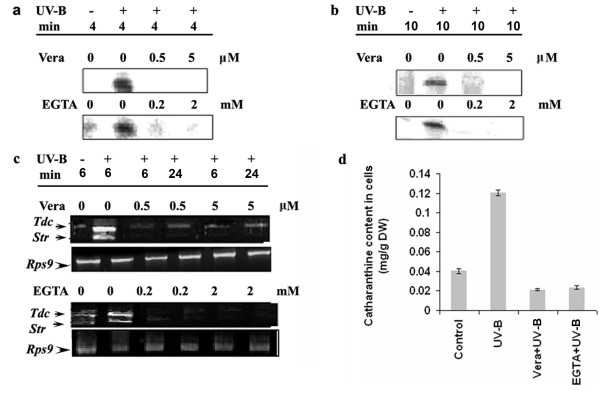
Effect of verapamil (Vera) and EGTA on UV-B-induced CDPK activity (**a**), MBPK activity (**b**), *Tdc *and *Str *gene expression (**c**) and accumulation of catharanthine (**d**) in cell suspension cultures of *C. roseus*. Six-day-old cell suspension cultures were pre-incubated with verapamil or EGTA at concentrations indicated followed by 5 min of UV-B irradiation. Other details are as in the legend to Figure 5.

Changes in membrane permeability and the resulting ion fluxes mainly Ca^2+ ^and H^+ ^influx, and K^+ ^and Cl^- ^efflux, are among the most rapid responses of plant cells to elicitation [23,24] Among these ion fluxes, the influx of Ca^2+ ^play an important role in transduction of the elictor signal and for elicitor-induced accumulation of plant secondary metabolites [25]. To assess whether Ca^2+ ^influx is involved in the UV-B-induced signaling pathway leading to catharanthine accumulation, the *C. roseus *cultured cells were treated with a specific calcium chelator EGTA prior to the UV-B irradiation and the UV-B induced responses were examined. Because EGTA is not likely to enter the cell, we expected it to make extracellular Ca^2+ ^at least partially unavailable for entering the cytoplasm by chelation. Pre-treatment with EGTA reduced the UV-B stimulated MBPK and CDPK activities to a very large extent indicating EGTA blocked the UV-B responses (Figure 6a and 6b). The level of the *Tdc *and *Str *transcripts and catharanthine content in the UV-B irradiated cells also reduced gradually as the EGTA concentration increased (Figure 6c and 6d). The involvement of calcium in the UV-B induced signaling pathway leading to catharanthine accumulation was further confirmed by studying the effect of verapamil, the plasma membrane calcium channel blocker, on the UV-B-induced responses. As shown in Figure 6a and 6b, verapamil inhibited the UV-B-induced MBPK and CDPK activities to a significant extent. UV-B-induced accumulation of *Tdc *and *Str *transcripts also decreased upon treatment with verapamil (Figure 6c). The catharanthine content in verapamil pre-treated cells also reduced significantly (Figure 6d). These results indicate that UV-B-induced catharanthine accumulation requires elevated levels of cytosolic calcium, and this increase is brought about by an influx of calcium from extracellular space.

### Role of protein phosphorylation in UV-B induced responses in *C. roseus *cells

**Figure 7:**
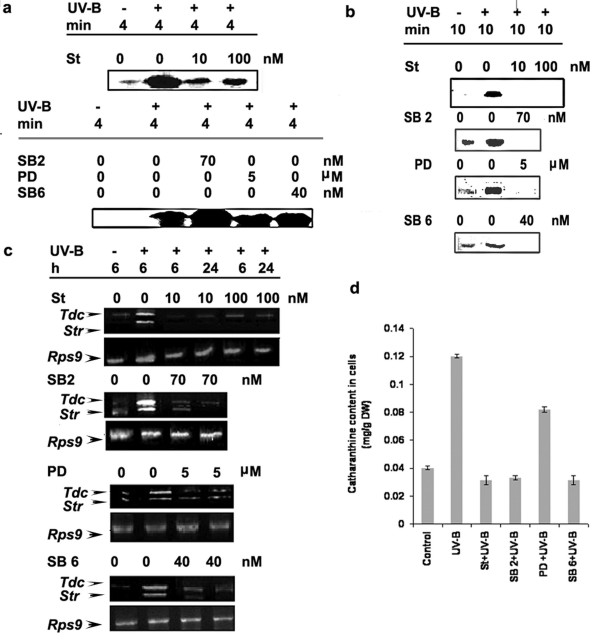
Effect of protein kinase inhibitor and MAPK cascade specific inhibitors on UV-B-induced CDPK activity (**a**), MBPK activity (**b**), *Tdc *and *Str *gene expression (**c**) and accumulation of catharanthine (**d**) in cell suspension cultures of *C. roseus*. Six-day-old cell suspension cultures were pre-incubated with staurosporine (St), SB 203580 a P38 inhibitor (SB2), PD 98059, an ERKK inhibitor (PD) or SB 600125 a JNK inhibitor (SB6) at concentrations indicated followed by 5 min of UV-B irradiation. Other details are as in the legend to Figure 5.

Having established that the activation of a 49-kDa MBPK and 55-kDa CDPK was induced by UV-B irradiation of *C. roseus *cells (Figs 3 and 4), we used this property in combination of inhibitors of protein kinases to assess possible involvement of these kinases in UV-B signaling pathway leading to catharanthine accumulation. The *C. roseus *cells were treated with inhibitors of protein kinases and the UV-B-induced responses, *viz*., MBPK and CDPK activities, *Tdc *and *Str *transcript accumulation and catharanthine content were examined. Staurosporine, a potent inhibitor of serine-threonine kinases, SB 203580, an inhibitor of P38 class of MAP kinase, PD 98059, an inhibitor ERKK class of MAPKK and SB 600125, an inhibitor of Janus kinases were used to assess the role of protein phosphorylation in UV-B responses. As shown in Figure 7a and 7b, staurosporine, SB 203580, PD 98059 and SB 600125 treatments at the concentrations tested completely abolished the UV-B-induced MBPK activity whereas the UV-B-induced CDPK activity could not be completely inhibited by staurosporine and was not inhibited by SB 203580, PD 98059 and SB 600125 pretreatments of the cells. The inhibitory effect of staurosporine on both MBPK and CDPK activities indicates a common mechanism of action of the inhibitor on these protein kinases, as both of them belong to the family of serine-threonine kinases. As expected, inhibitors of the MAPK cascade only inhibited the UV-B-induced MAPK-like MBPK activity, but not CDPK activity. We next examined the accumulation of *Tdc *and *Str *mRNA's in protein kinase inhibitor treated cells by reverse transcription polymerase chain reaction (RT-PCR). As shown in Figure 7c staurosporine, SB 203580, PD 98059 and SB 600125 inhibited UV-B-induced *Tdc *and *Str *transcript accumulation. In a similar fashion, UV-B-induced catharanthine production was significantly decreased by the above-mentioned inhibitors (Figure 7d) indicative of the implication of MBPK and CDPK activities in elicitation of UV-B induced catharanthine biosynthesis. The data obtained by immunoprecipitaion experiments and with the use of MAPK cascade specific inhibitors suggests the involvement of a putative MAPK in response to UV-B.

**Figure 8:**
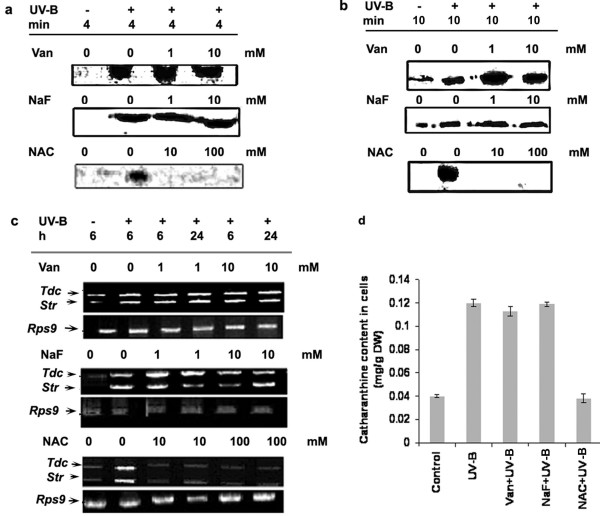
Effect of phosphatase inhibitor and phosphatase thiol group protector on UV-B-induced CDPK activity (**a**), MBPK activity (**b**), *Tdc *and *Str *gene expression (**c**) and accumulation of catharanthine (**d**) in cell suspension cultures of *C. roseus*. Six-day-old cell suspension cultures were pre-incubated with orthovanadate (Van), sodium fluoride (NaF), N-acetyl cysteine (NAC) at concentrations indicated followed by 5 min of UV-B irradiation. Other details are as in the legend to Figure 5.

As protein phosphatases antagonize the activity of protein kinases, we tested whether pre-treatment of cells with protein phosphatase inhibitors would show the opposite effect on the UV-B-induced responses. Interestingly, the addition of orthovanadate, a known inhibitor of tyrosine phosphatases [26] or sodium fluoride, a compound reported to strongly inhibit serine-threonine phosphatases [27], stimulated only the UV-B-induced MBPK activity at 1 and 10 mM concentrations substantially above the UV-B treated activity while that of CDPK activity remained unaffected (Figure 8b and 8a). The pretreatment of cells with orthovandate and sodium fluoride did not substantially increase the CDPK activity over and above the UV-B treated cells. To further test the role of protein phosphatases in the UV-B-induced protein phosphorylation activities, we used NAC, which is known to protect the thiol group of phosphatases from inactivation [26]. Pretreatment of cells with NAC inhibited the UV-B-induced MBPK and CDPK activities at 10 and 100 mM concentrations tested (Fig 8a and 8b). As shown in Figure 8c, pretreatment with orthovanadate or NaF did not increase the transcripts of *Tdc *and *Str *beyond the levels seen in cells irradiated with UV-B alone; however, NAC, on the other hand, decreased the UV-B-induced accumulation of *Tdc *and *Str *transcripts. At alkaloid level, we found that catharanthine accumulation in the *C. roseus *cells was greatly increased by UV-B irradiation (Figure 8d). Pretreatment of orthovanadate or sodium fluoride had no significant effect on the accumulation of catharanthine over and above the cultured *C. roseus *cells irradiated with UV-B alone. NAC had an overall inhibitory effect on the UV-B-induced *Tdc *and *Str *transcript levels as well as the catharanthine accumulation. NAC apart from protecting phosphatases from inactivation is also a potent inhibitor of ROS production. The results shown in Figure 2 as well as Figure 8 indicate that the UV-B signaling involves both ROS production and inactivation of phosphatases.

## Discussion

Several studies have demonstrated the involvement of signal components, such as receptors, Ca^2+ ^influx, medium alkalinization, oxidative burst, and protein kinases and phosphatases in responses to elicitors for enhanced production of secondary metabolites via increased transcription of relevant genes [8]. It has been shown earlier in *C. roseus *that the abiotic elicitor UV-B induces the formation of dimeric TIAs, and *Tdc *and *Str *mRNA accumulation [13]. There is also evidence that nuclear factor GT-1 function in the regulation of *Tdc *gene expression by UV light in *C. roseus *[14]. However, the UV-B signaling pathway that regulates activity of transcription factor GT-1 leading to *Tdc *gene expression is still obscure. In the present study, we present evidence for involvement of a putative receptor(s), calcium, reactive oxygen species, Ca^2+^-dependent protein kinase, and a putative MAPK in UV-B signaling and transcriptional activation of *Tdc *and *Str *genes and catharanthine biosynthesis in *C. roseus *cells.

Based on suramin interference with the binding of systemin to its cell surface receptor and UV-B responses in *L. peruvianum *cells [17] we used suramin to assess the involvement of a cell surface receptor in UV-B-induced expression of TIA biosynthetic genes. The results shown in Figure 1, 2 and 5 show that the UV-B-induced medium alkalinization, ROS production, CDPK and MBPK activities, *Tdc *and *Str *gene expression, and accumulation of catharanthine were all inhibited by suramin. Suramin *per se *is not known to affect medium alkalinization directly but acts via a receptor [17]. This suggested that suramin acts upstream of the afore-mentioned UV-B-induced responses and the UV-B-induced TIA biosynthesis. The inhibitory effect of suramin on the UV-B responses supports role of a putative cell surface receptor in UV-B signal pathway for the enhancement of *Tdc *and *Str *mRNA and catharanthine accumulation in the *C. roseus *cells.

We used a Ca^2+ ^chelator; EGTA, and Ca^2+ ^channel blocker, verapamil to investigate the role of Ca^2+ ^in UV-B induced responses. Both the treatments blocked the UV-B-induced stimulation of MBPK and CDPK activities and the UV-B-induced accumulation of *Tdc *and *Str *mRNAs, and catharanthine. Because EGTA and verapamil are unlikely to enter cells, and verapamil blocks the Ca^2+ ^channels localized in the plasma membrane [28,29], our data indicate that the influx of Ca^2+ ^from extracellular medium is required for the transduction of the UV-B signal, and that UV-B may influence the activity of the Ca^2+ ^channels. Our study does not rule out the possibility of mobilization of calcium from intracellular compartments such as endoplasmic reticulum, golgi body and vacuole. Ca^2+ ^signaling involves parallel and/or sequential use of different sources of Ca^2+ ^and different channels in different sub-cellular locations. It was demonstrated in tobacco cells that hypo-osmotic shock stimulates Ca^2+ ^influxes in a sequential manner, deriving first from external and then internal Ca^2+ ^stores and that these influxes are mediated by Ca^2+ ^channels [30]. Thus, the present study provides evidence that Ca^2+ ^serves as a second messenger in UV-B signal transduction involving activation of genes involved in TIA biosynthesis.

Our results also show that UV-B activated the generation of ROS in *C. roseus *cells (Figure 2a). The generation of ROS via an oxidative burst was shown to be induced by variety of elicitors, such as yeast elicitor on tobacco [31,32], chitin oligosaccharides in tomato [33], fungal oligosaccharides in red clover roots [34], and fungal elicitors in spruce [35] and parsley cell suspensions [36]. Using NAC, Ca^2+ ^channel blocker and broad range of kinase inhibitor staurosporine, we showed that protein phosphorylation and an increase in intracellular calcium levels are required for the UV-B induced activation of ROS production. The MAPK cascade inhibitors however had no effect on the production of ROS indicating the ROS production occurs upstream of MAPK cascade activation. The most likely source of UV-B-induced ROS production in *C. roseus *is a membrane-bound NADPH oxidase complex, which uses molecular oxygen to make superoxide [37]. In *Arabidopsis *suspension cells, a homologue of the catalytic subunit of the mammalian NADPH oxidase complex was shown to be responsive for ROS accumulation in response to bacterial protein elicitor harpin [38]. It has been shown that protein phosphorylation is needed for the production of ROS in potato tubers, spruce and tobacco cells [39]. The inhibitory effects of the protein kinase inhibitor staurosporine and Ca^2+ ^channel blockers on UV-B-induced ROS production in the *C. roseus *cells (Figure 2b) support the fact that a calcium-dependent protein kinase is involved in the UV-B induction of ROS production. There are a few reports that CDPK activates NADPH oxidase [40-43]. It remains to be determined whether UV-B-induced ROS are generated via induction of a NADPH oxidase activity by CDPK.

The phosphorylation and dephosphorylation of proteins have been thought to play a key role in the transduction of elicitor signals in plant cells. The data shown here indicated that irradiation of *C. roseus *cells with UV-B light strongly activates a 49 kDa putative MAPK and the activation of the 49 kDa putative MAPK in response to UV-B was associated with tyrosine phosphorylation on the kinase, a distinguishing feature of the large family of MAPK. We conclude that UV-B-activated 49 kDa putative MAPK is likely a member of the MAPK family. Our results (Figure 4) also suggest the involvement of Ca^2+^-dependent protein kinase (s) or Ca-CaM (calmodulin)-dependent protein kinase (s) in the UV-B response. MAP kinases, members of a group of serine/threonine protein kinases are important transducers of intracellular signals via protein phosphorylation that is initiated by various extracellular stimuli, and they are involved in proliferation, differentiation and responses to stress in animal and yeast cells [44]. Another notable aspect of this study is that staurosporine that has been used as an effective inhibitor of various protein kinases, completely inhibited both MAPK-like and CDPK activities (Figure 7a and 7b). It is noteworthy that pretreatments of specific synthetic inhibitors of MAPKs prevented stimulation of the UV-B-induced MAPK-like enzyme activity; however, no effects are observed for the CDPK activity (Figure 7a and 7b) suggesting that the activation of CDPK was relatively early as compared to the activation of putative MAPK. These data place MAPK downstream intermediaries in the cellular responses mediating catharanthine biosynthesis in response to UV-B and position CDPK upstream of MAPK. UV-B-mediated *Tdc*/*Str *gene transcription appeared dependent on activation of putative MAPK as well as CDPK pathway. The activity of a MAPK in cells is controlled through phosphorylation activation by its upstream kinases, MAPKK and MAPKKK, and dephosphorylation inactivation by its negative regulator, MAPK phosphatase/s. In this study, we showed that the UV-B-induced MAPK-like activity could be inhibited by PD98059, an inhibitor of ERKK (MAPKK), which similar to animal cells has no role to play in UV-B signaling. The results obtained using phosphatase inhibitors and NAC should be interpreted with caution because these inhibitors are not specific. NAC, for example is both a free radicle scavenger and phosphatase thiol group protector [26]. Phosphatase inhibitors, on the other hand, can affect the viability of cells at higher concentrations or can mediate an over all up-regulation in the kinase activities [45]. The reason we can attribute to absence of up-regulation in any of the UV-B-induced downstream activities in phosphatase inhibitors treated cells could probably due to the aberrational or toxic effect of these compounds on the entire cell homeostasis. In fact, treatment of cells with the inhibitors orthovandate or NAF alone activated many different kinases as assayed by MBP and H IIIS in gel phosphorylation assays (data not shown). The *Tdc *and *Str *activity and catharanthine accumulation in orthovanadate or NAF alone treated cells were again comparable to the UV-B alone treated cells (data not shown) demonstrating either imbalancing effects on cell homeostasis or that down-regulation of phosphatases alone are not the only event involved in the up regulation of the TIA pathway and other mechanisms do exist in regulation of TIA biosynthesis.

**Figure 9:**
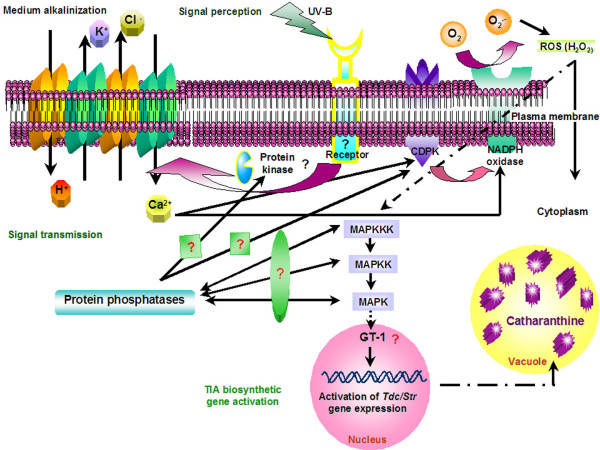
Proposed model for UV-B mediated signal transduction pathway leading to activation of the TIA pathway.

A tentative model for the UV-B signal flows, incorporating these present and previous findings, is illustrated in Figure 9. Upon perception of the UV-B light via a putative receptor, protein phosphorylation is required to induce influx of calcium via plasma membrane channels. This leads to a transient increase in cytosolic calcium levels, which is required for the subsequent activation of CDPK. Then, the activated CDPK would regulate the activation of NADPH oxidase in the plasma membrane and release ROS. Finally, downstream of ROS production, the UV-B-induced and -activated MAP kinases possibly participate in the activation of regulatory proteins such as GT-1 nuclear factor leading to transcriptional activation of TIA biosynthetic genes and enhanced production of catharanthine.

It has been earlier reported that yeast elicitor (YE) in *C. roseus *activates the octadecanoid pathway; leading to an increase in jasmonic acid (JA) levels via the activation of calcium influx and protein phosphorylation cascades [9]. JA induces the expression of the *ORCA3 *gene via post-translational modification which further interacts with the *Tdc *promoter and the YE and JA-responsive RV fragment of the *Str *promoter enhancing the gene expression [46-48]. YE reportedly also induce the expression of the zinc finger proteins, which by binding to specific elements within the promoter regions of *Tdc *and *Str *can repress its gene expression [49]. Similarly YE-induced CrBPF1 expression has been reported to be putatively involved in the regulation of *STR *via interaction with the BA region [50]. It would be interesting to understand whether the UV-B and YE-induced TIA pathway share common elements in signal transduction and also if UV-B utilizes any of the transcriptional initiators or repressors induced by YE in initiating the TIA pathway.

## Methods

### Chemicals

2', 7'- DCFH-DA, EGTA, heparin, histone IIIS, N-acetyl cysteine, phosphothreonine, phosphotyrosine, sodium fluoride, sodium orthovanadate and verapamil were purchased from Sigma Chemical Company, St. Louis, USA. Sodium β-glycerophosphate and sodium fluoride were from Hi-media Laboratories, India. Catharanthine and vindoline were obtained from Shanghai kangai biologicals, China. Staurosporine and suramin were obtained from MP Biomedicals, Germany. Monoclonal antibodies to phospho-serine and phospho-tyrosine, complete protease inhibitor cocktail and myelin basic protein were purchased from Upstate laboratories, U.S.A. SB 203580 (P38 inhibitor), PD 98059 (ERKK inhibitor) and SB 600125 (JNK inhibitor) were a kind gift from Prof. Anjali Karande, I.I.Sc, Bangalore.

### Cell culture and treatments of cells with UV-B and chemicals

*C. roseus *suspension-cultured cells were cultivated as described previously [51]. A three-ml of six-day-old culture in stationary growth phase was transferred aseptically to 35-mm petri plates and irradiated with UV-B (Minera lights, UVM 57, San Gabriel, California) directly, at a distance of 2.5 cm between the cultured cells and the lamp as described [51]. For chemical treatments, agonists or antagonists of effectors involved in other signal transduction pathways were diluted in water to the appropriate final concentrations, as indicated in figure legends from stock solutions prepared as described in Table 1. The cells were treated for 10 min with different chemicals (see Table 1) and subsequently irradiated with UV-B for 5 min, as indicated in figure legends. Control cultures were treated with an equivalent amount of water, ethanol or DMSO. Cells were harvested at the end of the treatment, immediately frozen in liquid N_2 _and stored at -80°C until use.

**Table 1:** Compounds used as agonists and antagonists to elucidate UV-B signal transduction pathway in *Catharanthus roseus *cultured cells

**Chemical**	**Working concentration (stock solution)**	**Effect/References**
EGTA	0.2 and 2 mM (0.2 M in water)	Calcium chelator [56]
N-acetyl cysteine	10 and 100 mM (10 M in water)	Scavenger of reactive oxygen species and protects thiol group of phosphatases from inactivation [26]
Sodium fluoride	1 and 10 mM (1 M in water)	Inhibitor of serine-threonine phosphatases [27]
Sodium orthovanadate	1 and 10 mM (1 M in water)	Inhibitor of tyrosine phosphatases [26]
Staurosporine	10 and 100 nM (10 μM in ethanol)	Broad range inhibitor of serine-threonine kinases
Suramin	0.1 and 1 mM (0.1 M in water)	Inhibits binding of growth factors to their receptors [17]
Verapamil	0.5 and 5 μM (0.5 mM in ethanol)	L-type calcium channel blocker [28, 29]
PD 98059 [2-(2-amino-3-methoxyphenyl) -oxanapthalen-4-one]	5 uM (0.5 mM in DMSO)	ERKK inhibitor [58]
SB 203580 [4-(4-flurop henyl)-2-(4-pyridyl) 1H imidazole]	70 nM (7 μM in DMSO)	P38 MAPK inhibitor [58]
SB 600125 ( anthrax-[1,9-cd]-6(2H)-one]	40 nM (4 μM in DMSO)	JNK inhibitor [59]

### Medium alkalinization response (AR) assay

To determine the UV-B-induced medium alkalinization, pH of the culture medium was measured from 0 to 120 min after 5 min of irradiation. UV-B-induced medium alkalinization response (AR) was calculated as the difference in pH between the untreated controls and the respective UV-B irradiated samples as described [15].

### Measurement of H_2_O_2 _production

H_2_O_2 _production was measured using cell permeable fluorescent probe 2', 7'-dichlorodihydroflurescein diacetate (DCFH-DA) by monitoring the increase in fluorescence by oxidation of DCFH to DCF (dichlorofluorescein) as described by Pauw et al. [37]. The 2.5 μM DCFH-DA was added to the cell suspension cultures immediately after UV-B irradiation. After UV-B irradiation for different time periods, the increase in intracellular H_2_O_2 _levels was measured by monitoring the increase in fluorescence after 15 min with 488-nm excitation and 525-nm emission wavelengths in a luminescence spectrometer (Perkin Elmer LS50B). To identify the events that inhibit the UV-B induced H_2_O_2 _production, various inhibitors were added for 10 min prior to 5 min-UV-B radiation.

### Preparation of the cell extract

Treated cell suspensions were collected by centrifugation, frozen separately in liquid nitrogen, and stored at -80°C until further use. Samples were thawed to 4°C and ultrasonicated (30 % amplitude, 15 pulses) in a buffer containing 50 mM HEPES-KOH pH 7.6, 2 mM DTT, 1 mM EDTA, 1 mM EGTA, 20 mM β-glycerophosphate, 20 % glycerol, 1 mM Na_3_VO_4_, 1 mM NaF and one tablet of complete protease inhibitors (Upstate) per 50 ml of buffer solution (EDTA and EGTA were excluded for calcium dependant kinase assays). Homogenates were centrifuged at 12,000 rpm at 4°C for 25 min. The supernatant was used immediately as a source of total soluble proteins to determine the activities of CDPK and MAPK. The total protein in the supernatant was estimated by the method of Bradford [52] using BSA as a standard.

### Protein kinase assays

Total soluble proteins extracted from *C. roseus *cells were assayed for CDPK and MBPK substrate phosphorylation activities according to the method of Putnam-Evans et al. [53] with slight modifications. Equal amounts of protein were taken and reactions were carried out in a total reaction volume of 30 μl kinase assay buffer (25 mM Tris pH 7.5, 5 mM MgCl_2_, 1 mM EGTA, 1 mM DTT and 2 μCi γ^32^P ATP for MAPK assay or in a buffer containing 25 mM Tris pH 7.5, 200 μM CaCl_2_, 10 mM MgCl_2 _and 2 μCi γ^32^P ATP for CDPK assay) for 30 min at room temperature. Substrate phosphorylation assays were done by adding 50 μg of myelin basic protein (MBP) or histone IIIS (HIIIS), respectively, to the same reaction buffer as mentioned above. The reaction was terminated by addition of electrophoresis sample loading buffer. After electrophoresis on 12 % SDS-polyacrylamide gels, the phosphorylated MBP and HIIIS were visualized by autoradiography.

CDPK and MBPK activities were determined by in-gel kinase assays using histone IIIS and myelin basic protein as substrates, respectively as described previously [41].

For immune complex kinase activity assays, MBPK and CDPK were immunoprecipitated using monoclonal anti-phosphotyrosine antibody and monoclonal anti-phosphoserine antibody, respectively as described by Stratmann and Ryan [54]. For immunoprecipitation, soluble proteins (200 μg) that had been made up to a total volume of 100 μl with immunoprecipitation buffer (10 mM Tris, pH 7.5, 150 mM NaCl, 1 mM EDTA, 1 mM EGTA, 1 mM Na_3_VO_4_, 1 mM NaF, 10 mM β-glycer0phosphate, 1 % [w/v] Triton X- 100, 2 mM DTT and one tablet of complete protease inhibitors per 50 ml of buffer solution) were incubated in a 1.5 ml eppendorf tube with 5 μg of monoclonal anti-phosphotyrosine or anti-phosphoserine antibody for 2 h at 4°C. For CDPK assay the same immunoprecipitation buffer was used without EDTA and EGTA. For reactions with competitor phosphoaminoacids, antibodies were preincubated for 30 min at room temperature with 1 mM of the phosphoaminoacid. Approximately 25 μl packed volume of recombinant protein A, immobilized on agarose, was added, and incubation continued for another 2 h at 4°C. The immunoprecipitated MBPK and CDPK were pelleted by centrifugation at 12,000 g for 10 min and washed two times with immunoprecipitation buffer. The samples were boiled for 2 min and separated by electrophoresis on 10 % SDS gels with MBP or H IIIS, respectively and in-gel kinase assays were done as described above.

### RNA isolation and RT-PCR analysis

Total RNA from cells of *C. roseus *was isolated using the Qiazol reagent (Qiagen Inc. Germany) following the manufacturer's instructions. The RNA samples were quantified by spectrophotometry at 260 and 280 nM (A260/A280 ~2.0; A260 = 40 μg RNA/ml) and visual inspection in agarose gels. DNA was removed from total RNA samples by treatment with RNase-free DNase I. Reverse transcription was carried out in a 20 μl reaction containing 1 μg of total RNA, 5 μg oligo d(T)_16–18 _primer, MuMLV reverse transcriptase (40 U), RNasin (20 U), 0.5 mM dNTPs and MuMLV reverse transcriptase reaction buffer (250 mM Tris-HCl, pH 8.3, 250 mM KCl, 20 mM MgCl_2 _and 50 mM DTT) at 37°C for 1 h, and terminated by heating at 70°C for 10 min. After the RT reaction, the cDNA was subjected to PCR reactions. The following pairs for primers were used: 5'-TGTAGCCATGTCCAATTCTCCAGT-3', as the forward primer and 5'-ATAAACTCGTCCCGTCGAGTTAAG-3', as the reverse primer for tryptophan decraboxylase (*Tdc *M25151), 5'-TAAATCCATGATGGCAGTTTTCTT-3', as the forward primer and 5'-ACCCACAGAGCTATGGAAGAGAC-3', as the reverse primer for strictosidine synthase (*Str *X61932). One μl of the RT reaction was used for PCR in 20 μl containing 0.4 U of Taq DNA polymerase (Fermentas), 0.1 mM dNTP (Fermentas), 200 μM of each dNTP and 100 pM of each primer in a 1× reaction buffer. Reactions were amplified for a total of 15 cycles on the Minicycler (MJ Research PTC-150) using 94°C for denaturation (1 min), 55°C for annealing for *Tdc *and *Str *and 52°C for annealing for *Rps9 *(1 min) and 72°C for extension (1 min), following a further 5 min extension. The RT-PCR products were separated by electrophoresis on 1 % agarose gels, stained with ethidium bromide, and photographed under UV light using Alpha Imager 2200 (Alpha Innotech Corporation, San Leandro, CA). RT-PCR analysis of ribosomal protein 9 (*Rps9*) was used as control to check RNA integrity and accuracy of loading. The primers were: *Rps9*-forwad 5'-TTAGTCTTGTTCGAGTTCATTTTGTAT-3', and *Rps9*-reverse 5'-GAGCAAATTAACTCAATTGATAATTAAC-3', (*Rps9*, AJ749993). The RT-PCR products of the expected sizes 1.5, 1.2 and 0.63 kb respectively was obtained for *Tdc*, *Str *and *Rps9 *and their identity confirmed by sequencing.

### Quantification of catharanthine by HPLC analysis

The extraction of terpenoid indole alkaloids and quantification of catharanthine using HPLC were according to Schripseme and Verpoorte [55]. The amount of catharanthine was finally reported as mg g ^-1 ^DW (dry weight) cells.

## Abbreviations

AR: Alkalinization response. Ca^2+^: Calcium ions. CDPK: Calcium dependent protein kinase. DCFH-DA: 2', 7'- dichlorofluoresceine diacetate. EGTA: Ethylene glycol bis(2-aminoethylether)- N,N,N'N'-tetraacetic acid. ERKK: Extracellular regulated kinase kinase. FDA: Fluorescein acetate. Hep: Heparin. HPLC: High pressure liquid chromatography. H IIIS: Histone IIIS. JNK: Janus kinase. MAPK: Mitogen activated protein kinase. MAPKK: Mitogen activated protein kinase kinase. MAPKKK; Mitogen activated protein kinase kinase kinase. MBP: Myelin basic protein; MS: Murashige and Skoog medium. NAA: α-naphthaleneacetic acid. NAC: N-acetyl cysteine. NaF: Sodium fluoride. PD 98059: 2-(2-amino-3-methoxyphenyl)-oxanapthalen-4-one. ROS: Reactive oxygen species. RT-PCR: Reverse transcription and polymerase chain reaction. SB 203580: 4-(4-fluorophenyl)-2-(4-methyl sulphinylphenyl-5-(4-pyridyl) 1H imidazole. SB 600125: anthra [1,9-cd]pyrazol-6(2H)-one. St: Staurosporine. STR/*Str*: Strictosidine synthase. Sur: Suramin. TDC/*Tdc*: Tryptophan decarboxylase: TIA: Terpenoid indole alkaloid pathway. UV-B: Ultraviolet B radiation. Van: Sodium orthovanadate. Vera: Verapamil. ΔpH: difference in pH between control and treated.

## Authors' contributions

SR was involved jointly in conceiving the study, carrying out the experimental work and drafting the manuscript. CJB was involved in conceiving the study and was involved in drafting the manuscript or revising it critically and has given final approval of the version to be published. Both authors read and approved the final manuscript.
